# Metformin Clinical Trial in HPV+ and HPV– Head and Neck Squamous Cell Carcinoma: Impact on Cancer Cell Apoptosis and Immune Infiltrate

**DOI:** 10.3389/fonc.2018.00436

**Published:** 2018-10-11

**Authors:** Joseph M. Curry, Jennifer Johnson, Mehri Mollaee, Patrick Tassone, Dev Amin, Alexander Knops, Diana Whitaker-Menezes, My G. Mahoney, Andrew South, Ulrich Rodeck, Tingting Zhan, Larry Harshyne, Nancy Philp, Adam Luginbuhl, David Cognetti, Madalina Tuluc, Ubaldo Martinez-Outschoorn

**Affiliations:** ^1^Department of Otolaryngology Head and Neck Surgery, Thomas Jefferson University Philadelphia, Philadelphia, PA, United States; ^2^Department of Medical Oncology, Thomas Jefferson University Philadelphia, Philadelphia, PA, United States; ^3^Department of Pathology, Anatomy and Cell biology, Thomas Jefferson University Philadelphia, Philadelphia, PA, United States; ^4^Department of Dermatology and Cutaneous Biology, Thomas Jefferson University Philadelphia, Philadelphia, PA, United States; ^5^Department of Pharmacology and Experimental Therapeutics, Thomas Jefferson University Philadelphia, Philadelphia, PA, United States; ^6^Department of Neurological Surgery, Thomas Jefferson University Philadelphia, Philadelphia, PA, United States

**Keywords:** head and neck cancer, squamous cell carcinoma, metformin, tumor microenvironment, immune infiltrate, HPV, tumor metabolism

## Abstract

**Background:** Metformin, an oral anti-hyperglycemic drug which inhibits mitochondrial complex I and oxidative phosphorylation has been reported to correlate with improved outcomes in head and neck squamous cell carcinoma (HNSCC) and other cancers. This effect is postulated to occur through disruption of tumor-driven metabolic and immune dysregulation in the tumor microenvironment (TME). We report new findings on the impact of metformin on the tumor and immune elements of the TME from a clinical trial of metformin in HNSCC.

**Methods:** Human papilloma virus—(HPV–) tobacco+ mucosal HNSCC samples (*n* = 12) were compared to HPV+ oropharyngeal squamous cell carcinoma (OPSCC) samples (*n* = 17) from patients enrolled in a clinical trial. Apoptosis in tumor samples pre- and post-treatment with metformin was compared by deoxynucleotidyl transferase dUTP nick end labeling (TUNEL) assay. Metastatic lymph nodes with extra-capsular extension (ECE) in metformin-treated patients (*n* = 7) were compared to archival lymph node samples with ECE (*n* = 11) for differences in immune markers quantified by digital image analysis using co-localization and nuclear algorithms (PD-L1, FoxP3, CD163, CD8).

**Results:** HPV–, tobacco + HNSCC (mean Δ 13.7/high power field) specimens had a significantly higher increase in apoptosis compared to HPV+ OPSCC specimens (mean Δ 5.7/high power field) (*p* < 0.001). Analysis of the stroma at the invasive front in ECE nodal specimens from both HPV—HNSCC and HPV+ OPSCC metformin treated specimens showed increased CD8+ effector T cell infiltrate (mean 22.8%) compared to archival specimens (mean 10.7%) (*p* = 0.006). Similarly, metformin treated specimens showed an increased FoxP3+ regulatory T cell infiltrate (mean 9%) compared to non-treated archival specimens (mean 5%) (*p* = 0.019).

**Conclusions:** This study presents novel data demonstrating that metformin differentially impacts HNSCC subtypes with greater apoptosis in HPV—HNSCC compared to HPV+ OPSCC. Moreover, we present the first *in vivo* human evidence that metformin may also trigger increased CD8+ Teff and FoxP3+ Tregs in the TME, suggesting an immunomodulatory effect in HNSCC. Further research is necessary to assess the effect of metformin on the TME of HNSCC.

## Introduction

Metformin is an oral biguanide anti-hyperglycemic and the most commonly prescribed medication for type 2 diabetes mellitus. Interestingly, data suggests that metformin may have antineoplastic properties as well. Retrospective epidemiologic analyses, while limited due to the number of confounders, have also shown a decreased incidence of HNSCC in patients taking metformin (1, 2). Diabetics treated with metformin have a cancer risk reduction of approximately 40% compared to diabetics not treated with metformin (3, 4). Other studies have also shown a reduction in the frequency of cancer with metformin use (5). Evans et al. reported that the risk of subsequent cancer diagnosis was reduced in patients with type 2 diabetes who received metformin (with an odds ratio of 0.85 for any metformin exposure versus no metformin exposure) (6).

Extensive preclinical data support the effectiveness of metformin as an antineoplastic agent (7–10). In HNSCC specifically, metformin inhibits proliferation of carcinoma cells and induces apoptosis *in vitro* and *in vivo*, in addition to reducing colony formation with cell cycle arrest *in vitro* (7). *In vitro* and *in vivo* animal models have also shown that metformin can prevent the conversion of premalignant oral lesions to squamous cell carcinoma. Metformin has been shown to reduce the size and numbers of oral tumors in mouse models treated with 4NQO and halt the progression of potential premalignant lesions (11). This study, by Vitale-Cross et al. not only showed a decrease in the total number of oral lesions in animals treated with metformin compared to control, but also showed almost complete absence of transformation to squamous cell carcinoma.

Additional preclinical data was recently published suggesting that there may be a synergy between immune-oncology agents and metformin. Scharping et al. demonstrated a substantial increase in response rate in the murine B16 melanoma model and MC38 colon adenocarcinoma model with combination therapy utilizing PD-1 axis inhibition in combination with metformin (12). PD-1 blockade alone in B16 resulted in no reduction of tumor burden and 20% reduction of tumor burden in MC38 mice. Metformin alone demonstrated no reduction in tumor burden. However, combination therapy of PD-1 blockade and metformin resulted in tumor regression in 70% of B16 mice and 80% of MC38 mice. Moreover, CD8+ effector T cells (Teff) showed increased production of effector cytokines in the combination therapy group, suggesting a direct impact on the immune TME. The interplay of the immune antitumor response and metabolism in the TME is an area of active investigation, as dysregulated metabolism in immune cells critically alters effector function. Immune cell metabolic exhaustion is believed to be one of the key factors contributing to suppressed cytotoxic CD8 effector function in the TME (13). Further, T cells undergo reversible anergy when they are placed in a low pH environment, characteristic of many cancers and particularly HNSCC (14, 15).

To further explore the impact of metformin in HNSCC, we performed a window of opportunity trial using metformin alone prior to definitive surgical resection in HNSCC patients. This study showed increase in the percent of samples showing caveolin1 (CAV1) expression in cancer associated fibroblasts (CAFs) from an average of 17–65% of the samples tested (16). This “rescue” of CAV1, a key regulator of mitochondrial metabolism, in CAFs suggests that metformin may induce a partial reversal of the metabolic phenotype of CAFs (17). We demonstrated a significant increase in tumor cell apoptosis in tumor cells after treatment with metformin by TUNEL (terminal deoxyribonucleotidyl transferase mediated dUTP-digoxigenin nick end labeling) assay (*p* < 0.001). Here we report results of novel sub-analyses on cancer cell apoptosis in HPV+ and HPV– groups and alterations in the immune TME mediated by metformin and an exploration of the impact of metformin treatment on the immune TME of involved lymph nodes.

## Materials and methods

### Subjects

**Figure 1:**
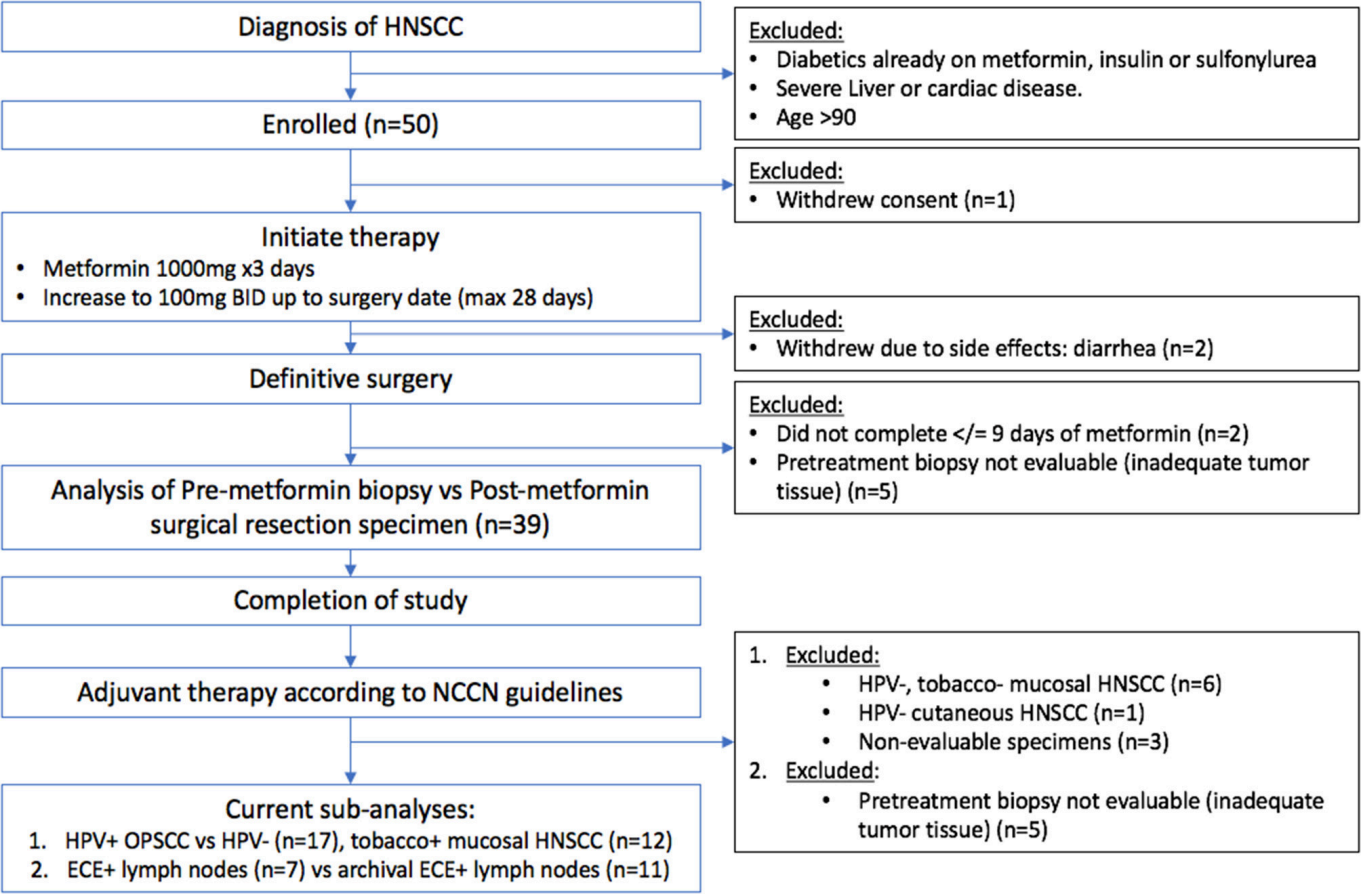
Consort flow chart for the clinical trial.

The metformin clinical trial specimens were primary tumor and lymph node samples obtained from a surgical trial in HNSCC patients. This study was carried out in accordance with the recommendations of Internal Review Board (IRB) of Thomas Jefferson University with written informed consent from all subjects. All subjects gave written informed consent in accordance with the Declaration of Helsinki. The protocol was approved by the Internal Review Board of Thomas Jefferson University. The trial was conducted with IRB approval and design, enrollment, and demographics are as described in detail elsewhere (16). (https://www.clinicaltrials.gov/ct2/show/NCT02083692). The flow for the trial is shown in Figure 1. Briefly, paired pre- and post-operative primary tumor tissue samples were compared for each patient. Patients included had a new, pathologically-confirmed diagnosis of HNSCC. Demographics are shown in Table 1. Enrolled patients received metformin titrated up every 3 days up to a standard anti-diabetic dose of 1000mg twice daily for up to 28 days prior to surgery with only specimens from patients with ≥9 days (range 9–24, mean 13.6 days) of therapy being analyzed. Patients received no additional cancer treatment while metformin was being administered. Of the 50 HNSCC patients enrolled, 39 completed the course of at least 9 days and had evaluable data and pre and post treatment specimens; of these 17 were HPV+ oropharyngeal squamous cell carcinoma (OPSCC) and 22 were HPV– HNSCC. Primary tumor samples were categorized according to HPV and smoking status. HPV status was demonstrated by p16 staining and subsequent *in situ* hybridization for HPV16 and 18. The pre-treatment and post-treatment specimens were compared by terminal deoxynucleotidyl transferase dUTP nick end labeling (TUNEL) assay for tumor cell apoptosis. For the current TUNEL comparison, HPV+ OPSCC samples (*n* = 17) were compared to HPV–, tobacco + HNSCC samples (*n* = 12). To assess immune elements of the TME, analysis of CD8, FoxP3, PD-L1, and CD163 staining intensity was carried out via digital image analysis using Aperio (Leica Biosystems, Wetzlar, Germany) co-localization and nuclear algorithms for assessment of intensity and quantification. FFPE lymph node specimens from patients on the trial with nodal metastasis demonstrating ECE (*n* = 7) were compared to archival FFPE ECE lymph node specimens (*n* = 11) from patients not receiving metformin either on trial or as a part of their medical record prior to surgery. Archival specimens were obtained with IRB approval.

**Table 1:** Demographics for clinical trial patients.

**Demographics**			
Age		61.7 (35–80)	
Gender		28 m/11 f	
Days on metformin		13.56 (9–24)	
**Anatomic subsite**			
Oral cavity	13 (33.3%)	**Oropharynx**	21 (53.8%)
Skin	1 (2.6%)	HPV+ OPSCC	17/21 (81.0%)
Larynx	3 (7.7%)	**Hypopharynx**	1 (2.6%)
**Staging (AJCC 2017)**			
***T*** **stage**		***N*** **stage**	
Tis	1 (2.6%)	N0	16 (41.0%)
T1	8 (20.5%)	N1	3 (7.7%)
T2	19 (48.7%)	N2a	5 (12.8%)
T3	3 (7.7%)	N2b	12 (30.8%)
T4a	8 (20.5%)	N2c	0 (0%)
T4b	0 (0%)	N3	3 (7.7%)
**Pathologic markers**		**Differentiation**	
ECE	10 (25.6%)	*in situ*	1 (2.6%)
Positive margins	4 (10.3%)	Well	3 (7.7%)
PNI	16 (41.0%)	Moderate	18 (46.2%)
LVI	14 (35.9%)	Poor	16 (41.0%)

*HPV, human papilloma virus; OPSCC, oropharyngeal squamous cell carcinoma; AJCC, American Joint Commission on Cancer; Tis, tumor stage in situ; ECE, extracapsular extension; PNI, perineural invasion; LVI, lymphovascular invasion*.

### Immunohistochemistry

Tissue sections (4 μm) for IHC analysis were dewaxed, rehydrated through graded ethanols, and antigen retrieval was performed on the Ventana Discovery ULTRA staining platform using Discovery CCI (Ventana cat#950-500) for a total application time of 64 min. Secondary immunostaining used a Horseradish Peroxidase (HRP) multimer cocktail (Ventana cat#760-500) and immune complexes were visualized using the ultraView Universal DAB (diaminobenzidine tetrahydrochloride) Detection Kit (Ventana cat#760-500). Slides were then washed with a Tris based reaction buffer (Ventana cat#950-300) and stained with Hematoxylin II (Ventana cat #790-2208) for 8 min. The CD8 antibody (CONFIRM Anti-CD8 SP57 Rabbit Monoclonal Primary antibody) and PD-L1 antibody (VENTANA PD-L1 (SP263) Rabbit Monoclonal Primary Antibody were obtained from Roche-Ventana, Tucson,AZ, USA). The FoxP3 (SP97) antibody was obtained from Spring Biosciences (Pleasanton, CA). The CD163 antibody (MRQ-26 mouse monoclonal antibody) was obtained from Cell Marque (Rocklin, CA, USA). Sections were counterstained with hematoxylin.

For quantification of apoptotic cells the TUNEL-based ApopTag Peroxidase *in situ* Apoptosis Detection Kit (Millipore Sigma Lifesciences, Burlington, MA) was used. The number of nuclei with TUNEL staining per high power field (hpf) was averaged over five HPFs in each specimen, and the mean was reported. For each sample, scores from two pathologists were averaged to yield a final score for statistical analysis.

Digital image analysis of CD8, FoxP3, CD163, and PD-L1 IHC was performed employing digital pathology with Aperio software as previously described (18). CD8 was utilized as a marker for Teff cells. FoxP3 was utilized as a marker for regulatory T cells (Tregs). CD163 was utilized as a marker for suppressive (M2) tumor associated macrophages (TAM). Programmed death ligand 1 (PD-L1) is highly expressed on cancer cells and some immune cells in the TME, such as TAMs. Briefly, tissue sections were scanned on a ScanScope™ XT at 40x, with an average scan time of 120 s (compression quality 70). Images were analyzed using the Color Deconvolution and the Colocalization Aperio Image Analysis tool. Areas of staining were color separated from hematoxylin counter-stained sections and the intensity of the staining was measured on a continuous 0 (bright white) to 255 (black) scale. For each marker, only the cells of highest intensity staining (digitally scored on a scale of 0–3+) were counted in the region of interest. Measurements from 5 hpf were taken and averaged to score each sample. The results are reported as % of nuclei/cells in a given region of interest with high intensity (3+) staining.

### Statistical analysis

Linear regression to assess strength of associations between continuous variables was performed. Significant *p*-values were considered < 0.05. TUNEL assay results were compared by multivariable robust linear mixed model with predictors (pre- vs. post-treatment) and HPV status as well as their interaction. CD8, FoxP3, CD-163, and PD-L1 IHC intensity was analyzed using Aperio as described above using a membrane staining algorithm using linear regression as above. Data were analyzed with R v3.5.0 (R-project.org).

## Results

### TUNEL apoptosis assay

**Figure 2:**
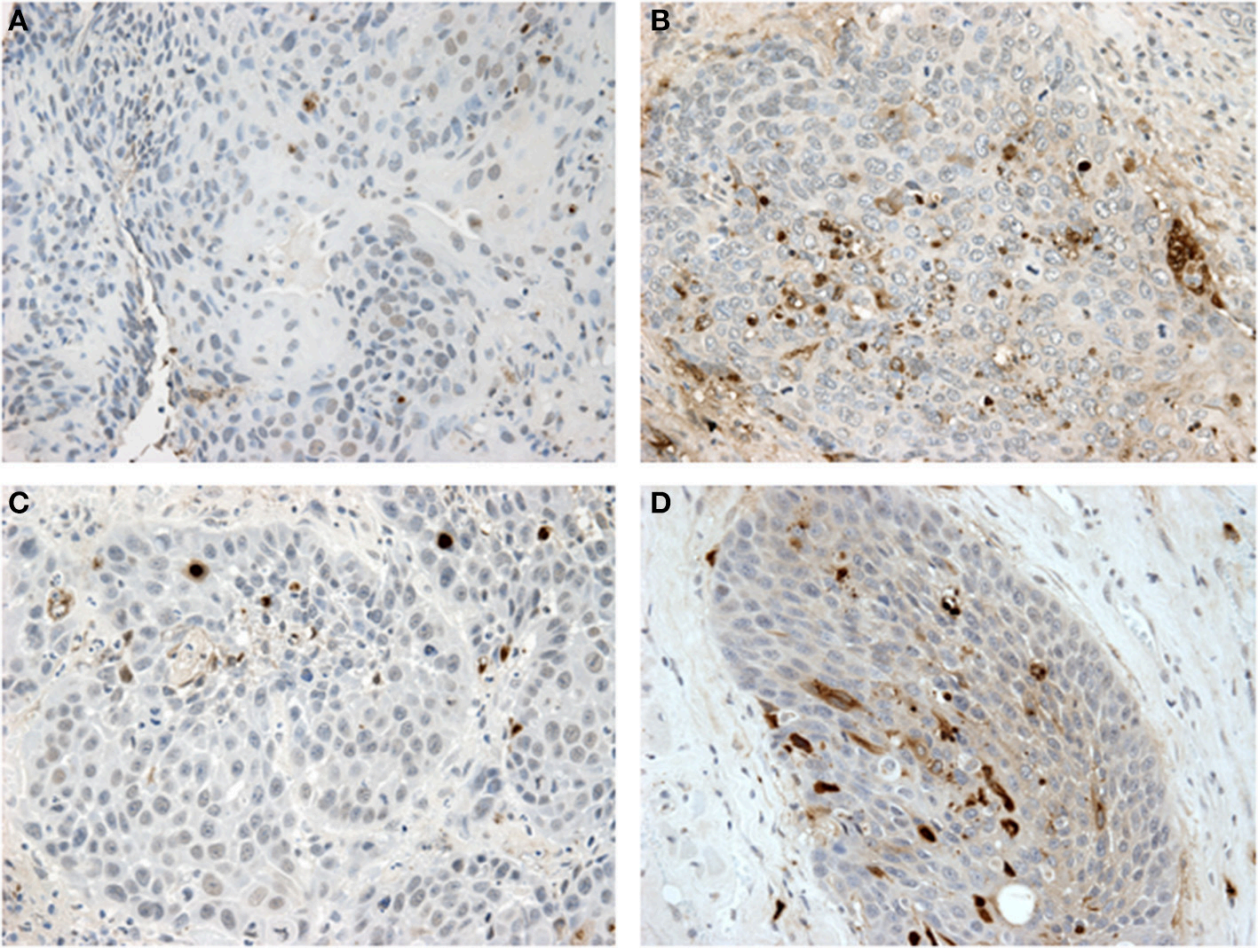
Tunel assay results: **(A,B)** show paired samples from an HPV—HNSCC specimen pre- and post-treatment with metformin showing a significant increase in apoptosis after metformin treatment. **(C,D)** show paired samples from an HPV+ OPSCC specimen also showing an increase in apoptosis after metformin treatment.

**Figure 3:**
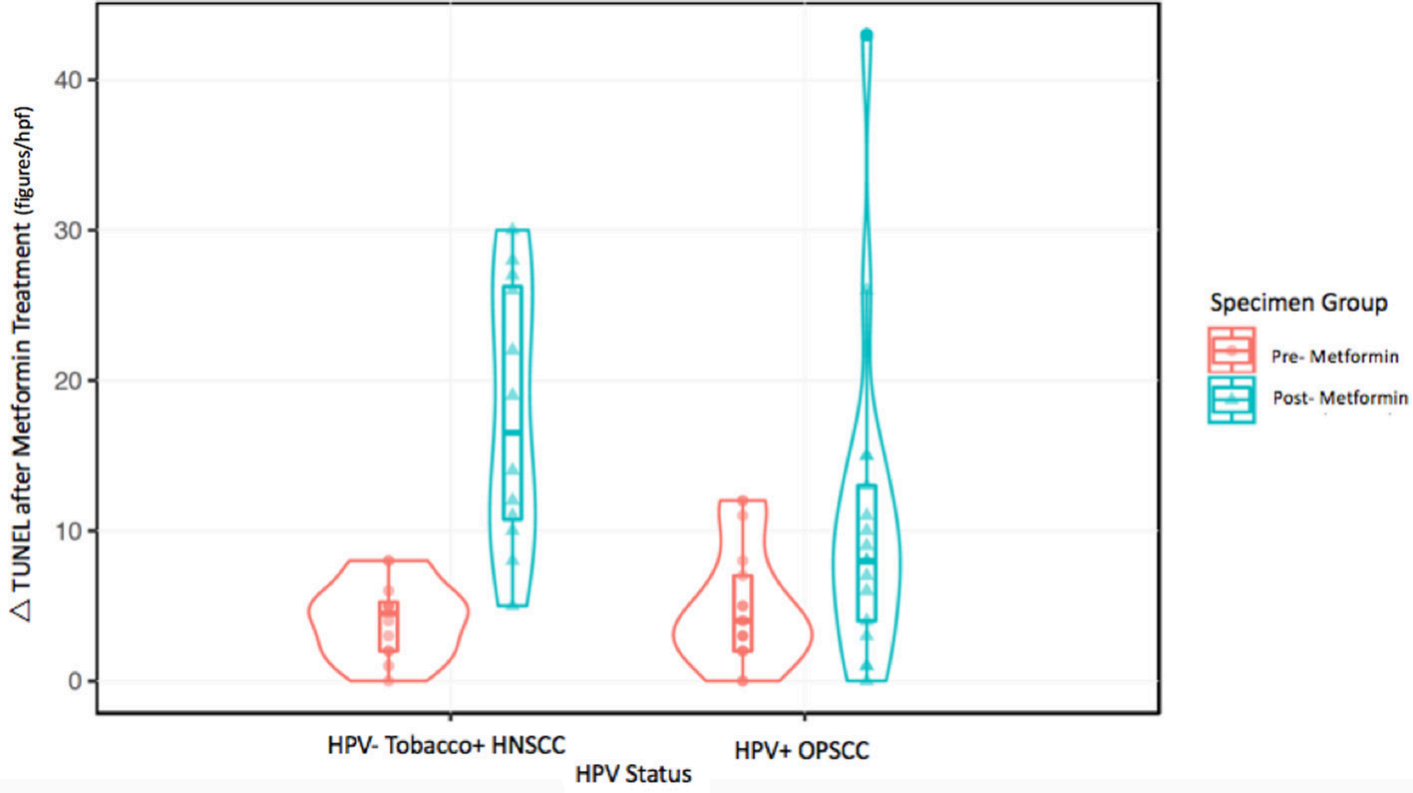
Violin plot for regression analysis of TUNEL data.

The metformin pre- and post-treatment groups were stratified and compared by tobacco and HPV status for analysis of tumor cell apoptosis in the primary tumor samples. Among these patients, there was a significantly greater increase in apoptosis with metformin treatment in HPV–, tobacco+ mucosal HNSCC (*n* = 12, mean Δ 13.7/hpf) compared to HPV+ OPSCC (*n* = 17, mean Δ 5.7/hpf) (*p* < 0.001) (Figures 1–3). Number of days treated with metformin for the two groups was similar (*p* = 0.92, *t*-test), with a mean 13.58 days for the HPV-tobacco+ group (range: 10–16 days) and a mean 13.71 days for the HPV+ group (range: 9 – 24).

### Immune infiltrate in node specimens

**Figure 4:**
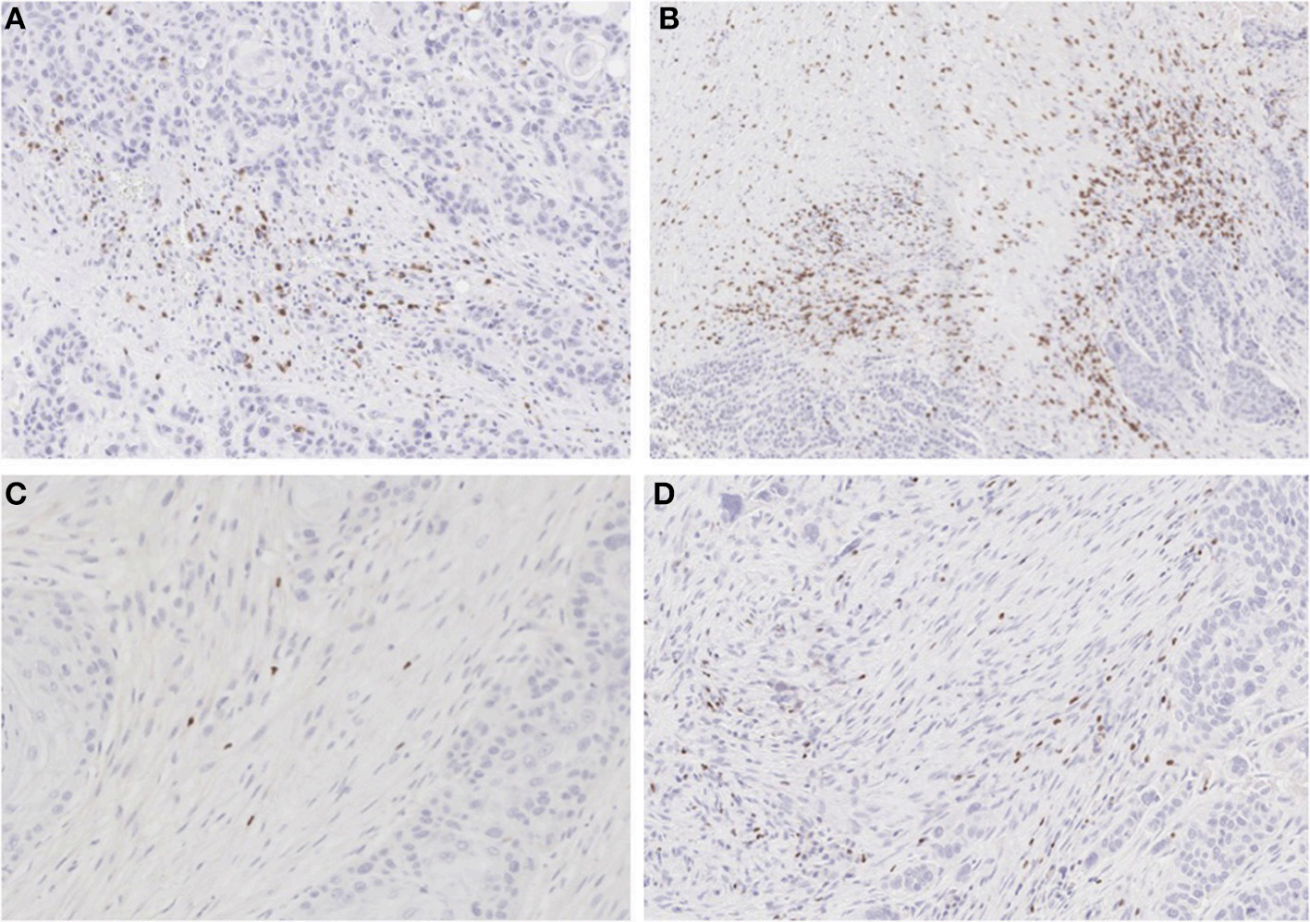
IHC for CD8 and FoxP3: **(A)** non-treated and **(B)** metformin treated samples stained for CD8 showing higher Teff cell count in the metformin treated specimens. **(C)** non-treated and **(D)** metformin treated specimens stained for FoxP3, showing a higher Treg count in metformin treated specimens.

**Figure 5:**
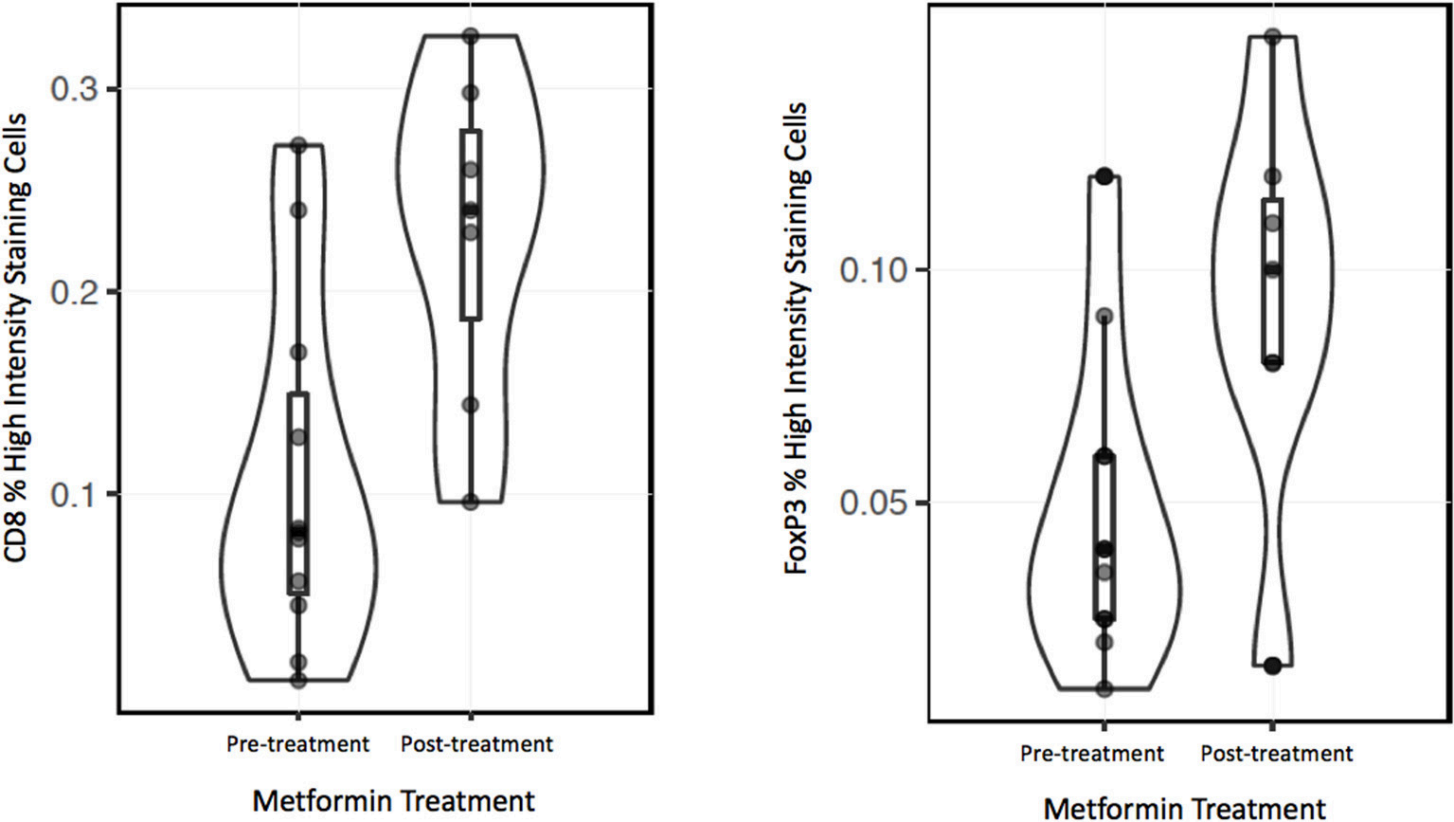
Violin plot for regression analysis of CD8 and FoxP3 IHC data.

To analyze the immune TME of the invasive tumor front, post-treatment lymph node specimens showing ECE were also compared to archival specimens with ECE. In a previous study, we demonstrated that the invasive front of lymph nodes with ECE is histologically similar to the invasive primary tumor front (19). The nodal samples were used to preserve limited pre-treatment primary tumor biopsy tissue for possible future analysis. Using the Aperio digital image analysis software, comparisons of staining intensity for CD8, FoxP3, CD163, and PD-L1 was made to quantify percentage of cells staining positive for each marker in the various compartments in the TME. In both groups, the CD163, FOXP3, and PD-L1 expression in the perinodal stroma at the site of invasion were significantly higher compared to regions with an intact lymph node capsule (*p* < 0.001) (Figures 4, 5). Further, these areas were similar at the invasive front between the metformin and non-metformin groups with respect to CD163 and PD-L1 suggesting that there was no significant change related to metformin treatment. Most interestingly, CD8 infiltrates were significantly greater in the tumor stroma at the invasive front of the metformin group (mean 22.8%) compared to the control (mean 10.7%) (*p* = 0.006). Moreover, FoxP3+ Tregs were also greater in metformin treated group (mean 9%) compared to the non-metformin treated group (mean 5%) (*p* = 0.019).

## Discussion

Here we present novel data on analysis of specimens from a clinical trial of metformin in HNSCC demonstrating a significantly increased cancer cell apoptosis in HPV– tobacco+ mucosal HNSCC compared to HPV+ OPSCC tumor samples after metformin treatment (*p* < 0.001, Figures 1, 2). This differential effect on the two tumor types is intriguing, but of unclear etiology. The mechanism by which metformin may exert its anticancer effects is not entirely understood, but it is known to impact cellular metabolism by multiple mechanisms, including inhibition of mitochondrial complex I, upregulation of AMP kinase and subsequent regulation of mTOR (20). Thus the effect on cancers may be attributed to the metabolic impact on the TME by metformin. In one study, HPV– HNSCC has been shown to be highly glycolytic, generate large amounts of lactate and have high levels of hypoxia, while HPV+ tumors demonstrated that effective utilization of glycolysis and oxidative phosphorylation (21). We previously demonstrated that HNSCC samples with high expression of the lactate exporter, monocarboxylate transporter 4 (MCT4), staining have a worse prognosis; high expression of MCT4 correlates with high glycolysis (22). Taken together, these data suggest differing metabolism between the two tumor types with higher reliance on glycolytic metabolism in HPV–, tobacco+ HNSCC. It is possible that metformin exerts a metabolic strain that is beyond the tolerance of the already highly glycolytic HPV– tumors. Yet, further research is required to test this hypothesis. Alternatively, the anticancer effects of metformin may be indirectly mediated through effects on other elements of the TME such as enhanced immune activity as seen in animal models of other tumor types (12).

Therefore, we hypothesized that the anticancer effects of metformin may also be due to an increased anti-tumor immune response. Dysregulated metabolism in the TME contributes to immune evasion through various mechanisms including impairment of tumor infiltrating T cell function, TAM function and checkpoint inhibition among other mechanisms (23, 24). Mitigation of this dysregulation in cancer may result in enhanced immune function. We analyzed specimens for alterations in key immune elements, including CD8, FoxP3, CD163, and PD-L1. For this exploratory analysis, we utilized lymph nodes with ECE as the invasive front at the site of ECE is a point of direct interaction with the surrounding tissue and stroma (19). We compared metformin treated specimens to matched archival specimens without metformin exposure. This group of ECE+ nodal specimens consisted of both HPV+ and HPV– lymph nodes from the trial, as the individual groups from the trial were too small to analyze separately. The archival samples were matched HPV+ and HPV– samples. No significant difference in PD-L1 or CD163 staining was noted in the cancer or tumor stroma of samples from patients that had received metformin or the controls.

However, there was a significant increase in the CD8+ T cell infiltrate in the tumor stroma at the invasive front in metformin treated patients compared to archival specimens. (Figures 4, 5) This finding represents the first clinical data to point to a link between a metabolic and immune altering mechanism for metformin. As described above, preclinical data in an immunocompetent C57BL/6 murine model with syngeneic MC-38 colon adenocarcinoma and B16 melanoma cancer cells demonstrates that metformin synergizes with PD-1 inhibition to substantially increase the antitumor effect of immunotherapy (12). Metformin inhibited oxygen consumption within the tumor, resulting in mitigation of hypoxia mediating enhanced effect of PD-1 inhibition. This preclinical data combined with evidence of clinical efficacy provides support to explore the combination of metformin with anticancer immunotherapy in clinical trials. Further, there is currently a clinical trial underway in HNSCC to study the impact of metformin on tissue hypoxia (NCT03510390).

Interestingly, we also identified an increase in FoxP3+ Tregs in the metformin treated group. This finding was unexpected in light of the increased CD8+ T cells, as typically Tregs are thought to be suppressive and thought to inhibit an effective anti-tumor response (25). However, a recent meta-analysis suggests that in HPV– HNSCC a higher infiltration of FoxP3+ Tregs correlated with an improved prognosis (26). There is little data on the effects of FoxP3+ Tregs in HPV+ OPSCC but there is a trend toward improved survival with higher infiltrate, and one study showed evidence that the patients with higher peripheral Treg counts had a better prognosis (27–29). Recent data based on analysis of The Cancer Genome Atlas (TCGA) showed that HPV– tumors are characterized by an immune-poor TME with lower overall infiltrate and that this finding correlates with a worse prognosis (30). In contrast HPV+ OPSCC is characterized by an immune rich TME, which has a better prognosis. An increase in immune infiltrate may correlate with improved anti-tumor response.

This study has several limitations, including sample size is limited for both the apoptosis assay and the immune IHC studies, although statistical significance was reached. The sample size in this study was too small to determine whether HPV+ OPSCC or HPV– HNSCC had differential changes in immune infiltrates for CD8+ Teff or FoxP3+ Tregs. Additionally, we studied lymph node specimens from subjects receiving metformin and controls for analysis of the immune markers but the status of these immune markers is unknown in the primary tumor specimens. The immune status of the TME of the lymph node or surrounding soft tissue may differ from that of the primary tumor; however, we would argue that the data regarding the immune TME of lymph nodes is also important. Further, studies must be undertaken to validate these results and to obtain mechanistic data on how metformin causes increased cancer cell apoptosis and the increased immune infiltrate and also whether these two effects are directly linked. Nevertheless, this study does present intriguing preliminary data directly from HNSCC clinical trial samples.

## Conclusion

Metformin resulted in a greater increase in apoptosis in HPV– tobacco+ HNSCC than occurred in HPV+ OPSCC. While the mechanism has yet to be clarified, metformin also appears to alter the immune TME with an increased infiltrate of CD8+ Teff and FoxP3 Tregs at the invasive tumor margin of lymph nodes with ECE.

## Author contributions

JC design of study, data accrual, and manuscript preparation. JJ, DW-M, MGM, AS, UR, LH, NP, AL, DC, UM-O design of study, manuscript prep and editing. MM, PT, DA, AK, DW-M data accrual and management. MT pathology oversight, manuscript preparation, and editing. TZ biostatistics oversight.

### Conflict of interest statement

The authors declare that the research was conducted in the absence of any commercial or financial relationships that could be construed as a potential conflict of interest.
